# Prostaglandin I2 IP Receptor Agonist, Beraprost, Prevents Transient Global Cerebral Ischemia Induced Hippocampal CA1 Injury in Aging Mice

**DOI:** 10.4172/2329-6895.1000174

**Published:** 2014-09-02

**Authors:** Hania Shakil, Sofiyan Saleem

**Affiliations:** 1 Hamdard College of Medicine and Dentistry, Hamdard University, Sharae Madinat Al-Hikmah, Karachi 74600, Pakistan; 2 Del E Webb Center for Neuroscience, Aging and Stem Cell Research, Sanford Burnham Medical Research Institute, La Jolla, CA 92037, USA

**Keywords:** Beraprost sodium, Carotid artery occlusion, Neurobehavior, IP receptor, Aging

## Abstract

Beraprost sodium is a new stable, orally active Prostaglandin I2 analogue. The aim of this study was to determine the effect of beraprost on cognitive dysfunction and locomotor impairment induced by bilateral common carotid artery occlusion in mice. We investigated the ameliorating effect of beraprost through PGI2 IP receptor by studying neurologic deficit assessment and T-maze testing in young and old male C57Bl/6 wild-type (WT) and IP receptor knockout (IP KO) mice following a 12 min bilateral common carotid artery occlusion (BCCAo) and 7 days of reperfusion. Beraprost reversed BCCAo induced cognitive impairment and neurological deficit in a dose dependent manner. Immunohistochemical studies showed attenuation of neuronal cell death, astrogliosis, microglial invasion, and myeloperoxidase (MPO) activity in both young and old WT mice after post treatment with beraprost. Moreover, after BCCAo, phosphorylated cAMP response element binding protein positive cell numbers were increased with beraprost treatment over vehicle treated controls. These results show that beraprost treatment attenuated cognitive dysfunction and neurological deficits induced by BCCAo, and suggest that this effect may be mediated by the neuroprotective effects of treatment.

## Introduction

Transient global cerebral ischemia remains a leading cause of death or deterioration in the quality of life in aging animals and humans [1,2]. An understanding of the pathology and mechanism of action of the ischemic damage should be useful in developing efficient therapeutic approaches [3,4]. Damage to neurons after ischemia/reperfusion brain injury is mediated through excessive overload of intracellular calcium (Ca^2+^) through activation of channels by excitatory amino acids, an aberrant increase in reactive oxygen species and pro-inflammatory cytokines [5,6].

Previous reports suggest that cyclooxygenase 2 (COX-2) plays a critical role in hippocampal CA1 injury after global cerebral ischemia [7]. Different prostaglandins such as prostaglandin (PG) E2, PG D2, PGF2α, PGI2, and thromboxane A 2 mediate COX-2 effects mainly through G-protein-coupled receptors, i.e., PGE2 EP (1–4), PGD2 DP (1–2), PGF2α FP, PGI2 IP and TXA2 TP receptors. Each of these receptors mediates different effects EP2, EP4, DP1 and IP receptor through an increase in cyclic AMP (cAMP) levels and EP1, FP, TP cause increase in Ca^2+^ mediated toxicity [8].

PGI2 is an arachidonic acid metabolite synthesized sequentially *via* cyclooxygenase and prostacyclin synthase (PGIS). In general, activation of IP-receptors induces vasodilation and inhibits leukocyte adhesion and platelet aggregation [9]. Beraprost is the first PGI2 analogue available as an oral formulation to protect against pulmonary arterial hypertension in humans [10]. Beraprost has been reported to protect against left carotid artery occlusion induced hippocampal CA1 brain injury in gerbils [11] and to prevent nephropathy via phosphorylation of cyclic AMP response element binding (CREB) protein [12], which is also involved in learning and memory and ischemic tolerance [13,14].

Here we have investigated the therapeutic effect of beraprost by measuring cognitive and motor deficits as well as neuronal death in young and old WT and IP KO mice subjected to a 12 min bilateral common carotid artery occlusion (BCCAo) and 7 days of reperfusion. In addition, we determined the degree of microglia activation and leukocyte infiltration as measured by MPO levels. We also quantified the phosphorylation of CREB, which interacts with the transcription co-activator CREB-binding protein to initiate the transcription and translation of CREB target genes, which are required for synaptic plasticity, learning and memory. This study highlights the therapeutic benefits of beraprost in young and old WT mice which correlates well with previous animals and human population studies [15–19]. On the other hand, the beraprost treatment showed no improvement in behavioral and histological outcomes after genetic deletion of PGI2.

IP receptor in young and old IP KO mice. Overall, this is a first report demonstrating that the beraprost mediates its therapeutic effect through downstream G-protein coupled IP receptor by restoring animal behavior functions and increasing neuronal cell survival and phosphorylation of CREB and decreasing astrogliosis, microglia invasion and neutrophil infiltration after 12 min of global cerebral ischemia and 7 d of reperfusion especially in old mice.

## Materials and Methods

### Experimental animals

This study was performed in accordance with the NIH guidelines for the use of experimental animals. All protocols were approved by the Sanford-Burnham Medical Research Institute Animal Care and Use Committee. The male young (2–3 months) and old (12–15 months) wild-type (WT) and IP receptor knock out (IP KO) C57BL/6 mice were received from Dr. Garret Fitzgerald were maintained and housed in our barrier facility, feed *ad libitum* and genotyped by polymerase chain reaction as described earlier [20]. Beraprost sodium is a water soluble compound purchased from Cayman chemicals.

### Experimental groups

Mice were randomly divided into eight groups: Vehicle (normal saline) and beraprost (25–100 μg/kg body weight per os) treated 4 h after reperfusion and every day up to 7 days in young and old ischemic WT and IP KO mice (n=8–10).

#### Induction of Global Cerebral Ischemia

BCCAo to induce ischemia, mice were anesthetized with 5% isoflurane and intubated with a small-animal respirator (Harvard, type 845, Harvard Inc., Holliston, MA, USA). The cerebral blood flow (CBF) was measured by laser Doppler flow cytometry (Perimed Inc, Ardmore, PA). A midline incision was made, and then the bilateral common carotid arteries were carefully isolated and occluded by artery clips. After 12 min, the clips were removed to restore cerebral blood flow and the incision was closed. The body temperature was maintained at 37°C with a heating pad throughout the procedure and recovery [13,21].

#### T Maze Spontaneous Alternation

T-maze spontaneous alternations were determined at day 7 of reperfusion as previously reported [13]. Briefly, mice were placed at the base of a T maze and were given the choice to explore either the right or left arm of the maze for ten consecutive trials. A choice was assumed to be made when the mouse stepped with all four paws into an arm. At that moment, the gate to that arm was closed and the animal was allowed to explore the arm for 5 s.

#### Open field test

Mouse motoric behavior testing was performed at day 7 of reperfusion using an open field scoring system, which measures each mouse’s gross locomotor ability inside a plastic tray (8×11×3 inches) according to a modified Tarlov scale. Locomotor activity scores ranged from 0 (flaccid or spastic paralysis) to 5 (normal walking) as previously described [13].

## Immunohistochemistry and Quantification

The animals were sacrificed at day 7 after behavior studies. The brains were harvested and fixed in 4% Paraformaldehyde. Paraffin-embedded brain sections were de-waxed in xylenes and rehydrated through graded alcohol solutions. Antigen retrieval was achieved by microwaving sections in citric acid (pH 6) for 5 min. Endogenous peroxidase activity was quenched by exposure to 10% hydrogen peroxide for 30 min. Sections were blocked for 1 h in 4% normal goat serum before being exposed to primary antibody overnight at 4°C. Anitbodies used were: NeuN (Millipore Chemicon, Billerica, MA), specific for neurons; anti-GFAP (Abcam, Cambridge, UK), specific for astrocytes; Iba 1 (Wako Chemicals USA, Richmond, VA), specific for microglia/macrophages; anti-MPO (Dako North America Inc, Carpinteria, CA), specific for neutrophils; anti-phosphorylated-CREB (Ser 133) (Cell signaling, Danvers, MA). Following overnight incubation at 4°C, sections were incubated in an appropriate secondary antibody (Vector Laboratories, Burlingame, CA) for 1 h at room temperature. Staining was visualized with the ABC-DAB system (Vector Laboratories). Positively immunostained cells in the whole hippocampal CA1 subfield of each section were quantified using the Aperio Scan Scope CS system (Aperio Technologies). The acquired digital images representing whole tissue sections were analyzed by applying the Spectrum Analysis algorithm package and Image Scope analysis software (version 9; Aperio Technologies).

## Statistical Analysis

GraphPad PRISM was used for all statistical tests. We performed a two way ANOVA for behavior tests and cell counts between the vehicle- and beraprost-treated young and old WT and IP KO experimental groups followed by Fisher’s Protected Least Significant Difference (PLSD) post hoc analysis. An alpha level of 0.05 from a Chi Squared test was used to reject the null hypothesis. Data are presented as mean ± standard error of the mean (s.e.m.), and a p value<0.05 was considered statistically significant.

## Results

We performed 12 min of global cerebral ischemia on test mice followed by treatment with either vehicle or beraprost (25–100 μg/kg body weight p.o.) given from 4 h into reperfusion then daily for up to 7 days. There was drop in CBF in young vehicle and beraprost (B-25, B-50 and B-100) treated C57Bl/6 WT groups (9.5 ± 0.6%; 9.0 ± 0.8%; 10.0 ± 1.0%; 9.8 ± 0.9% from baseline) as well as young vehicle IP KO and IP KO B-100 groups (8.7% ± 0.5%; 8.8% ± 0.9% from baseline).

**Figure 1:**
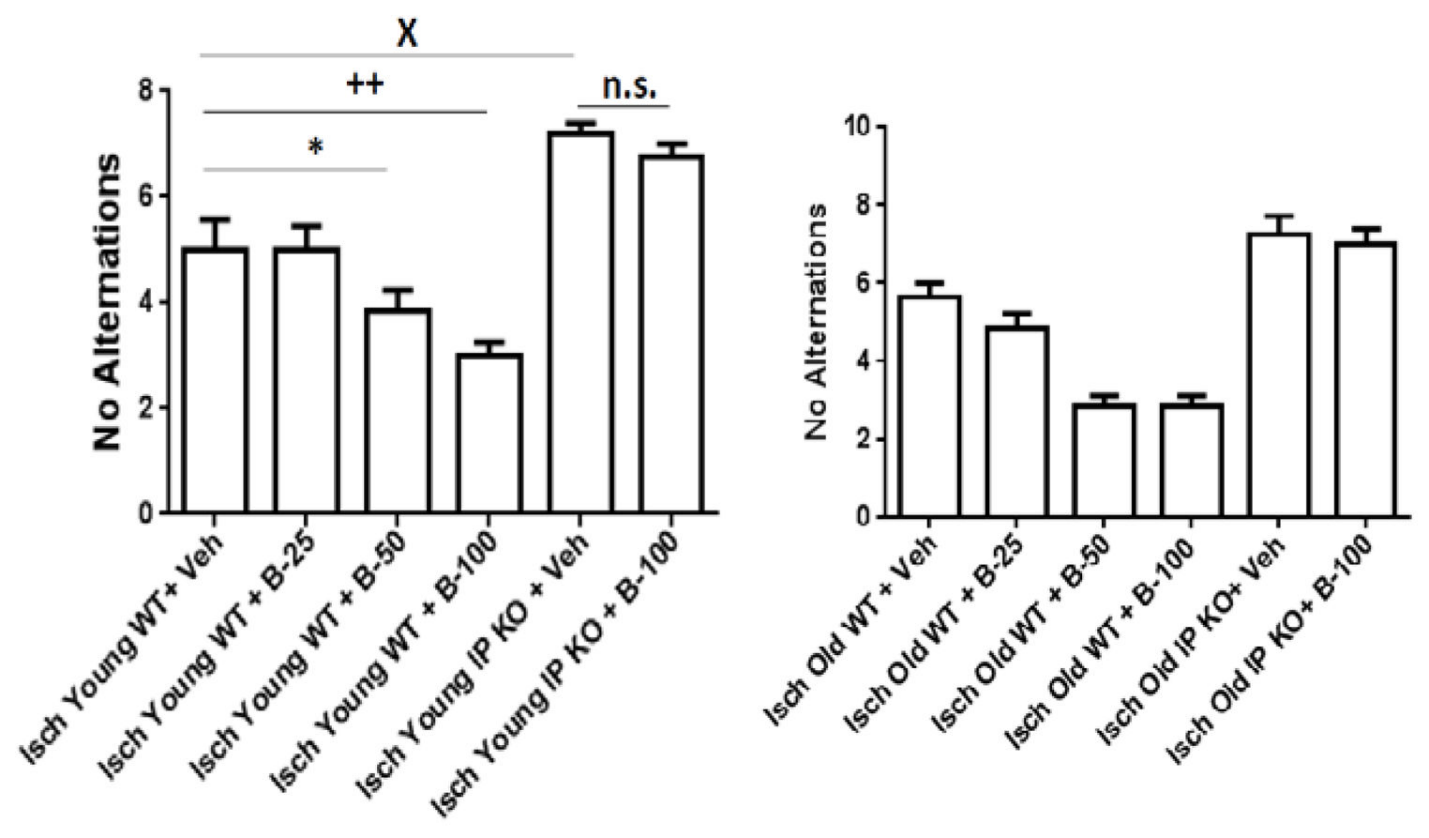
**A and B:** Behavioral recovery after beraprost treatment. 1A)Young and 1B) Old groups of WT and IP KO mice were subjected to 12 min global ischemia and the effects of treatment with Beraprost (25–100 μg/kg p.o. represented as B-25, B-50 and B-100) (vs. control vehicle [Veh]) 4 h after starting reperfusion were evaluated. After 7 d of reperfusion, recovery from the ischemic [32] insult was evaluated. “No Alternations” in the T-maze (10 trials) were recorded. (n=8–10 per group). *P<0.05; ++P<0.01; XP<0.05 vsIsch Young WT+Veh; **P<0.01, ++P< 0.01; XP<0.05 vsIsch Old WT + Veh; n. s.=not significant). Data are presented as mean ± s.e.m.

**Figure 2:**
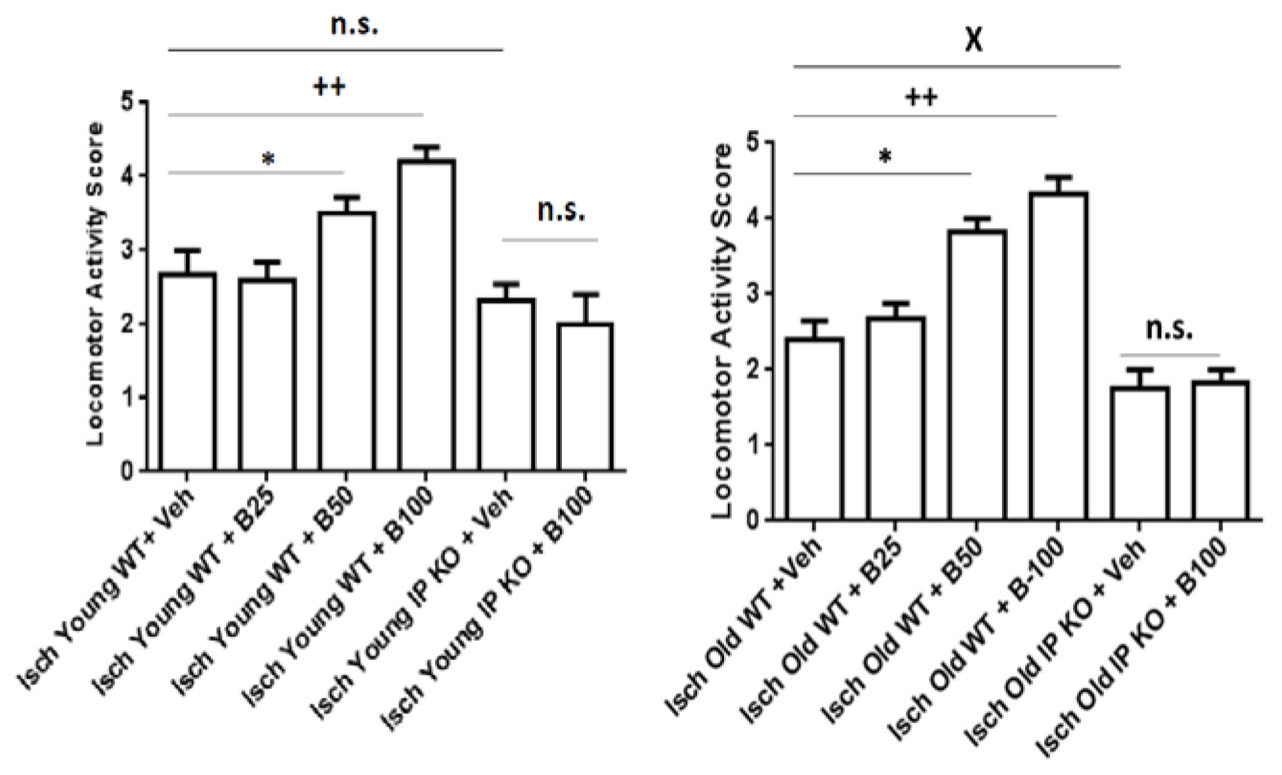
**A and B:** Locomotor activity score after beraprost treatment. 2A) Young and 2B) Old groups of WT and IP KO mice were subjected to 12 min global ischemia and the effects of treatment with Beraprost (25–100 μg/kg p.o. represented as B-25, B-50 and B-100) (vs. control vehicle [Veh]) 4 h after starting reperfusion were evaluated. Locomotor activity was assessed by a 5-point scale (n=8–10 per group). *P<0.05; ++P<0.01 vsIsch Young WT+Veh; *P<0.01, ++P<0.01; XP<0.05 vsIsch Old WT+Veh; n. s.=not significant). Data are presented as mean ± s.e.m.

**Figure 3:**
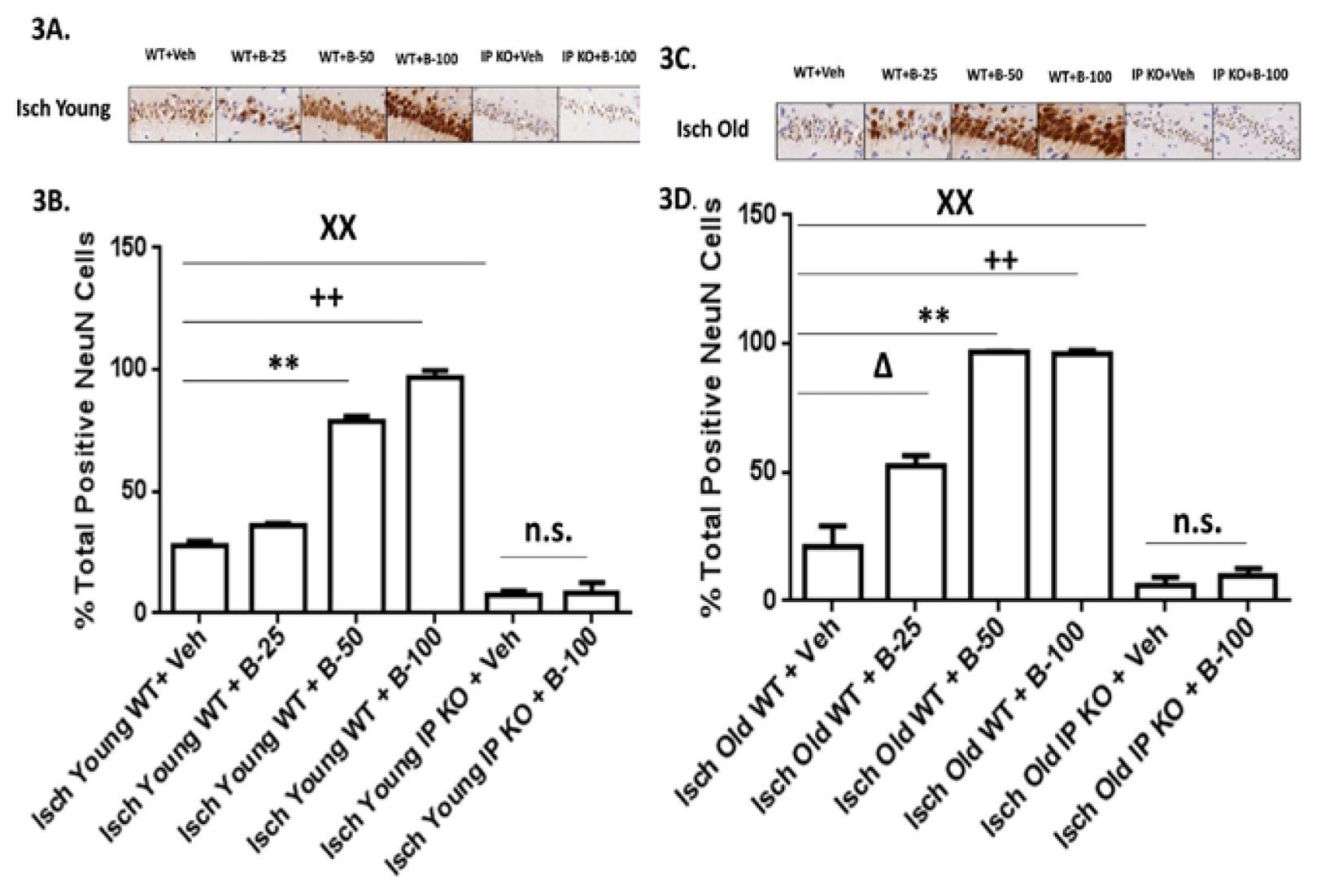
**A–D:** Beraprost treatment preserves hippocampal neurons. (3A and 3C). Representative images of brain sections show NeuN-immunostaining in Young and Old vehicle and beraprost treated (B-25, B-50 and B-100) WT and IP KO ischemic mice. (3B and 3D). Cell death in ischemic mice after beraprost treatment (B-25, B-50 and B-100) 4 h after reperfusion was determined by quantification of NeuN-positive cells in the hippocampal CA1 subfield on day 7 after ischemia, comparing ischemic Young and Old IP KO groups with their ischemic WT controls. **P<0.05, ++P<0.01, XXP<0.01 vsIsch Young WT+ Veh; P<0.05, **P<0.01: ++P<0.01, XXP<0.01 vsIsch Old WT+ Veh; n. s.=not significant. Data are presented as mean ± s.e.m.

**Figure 4:**
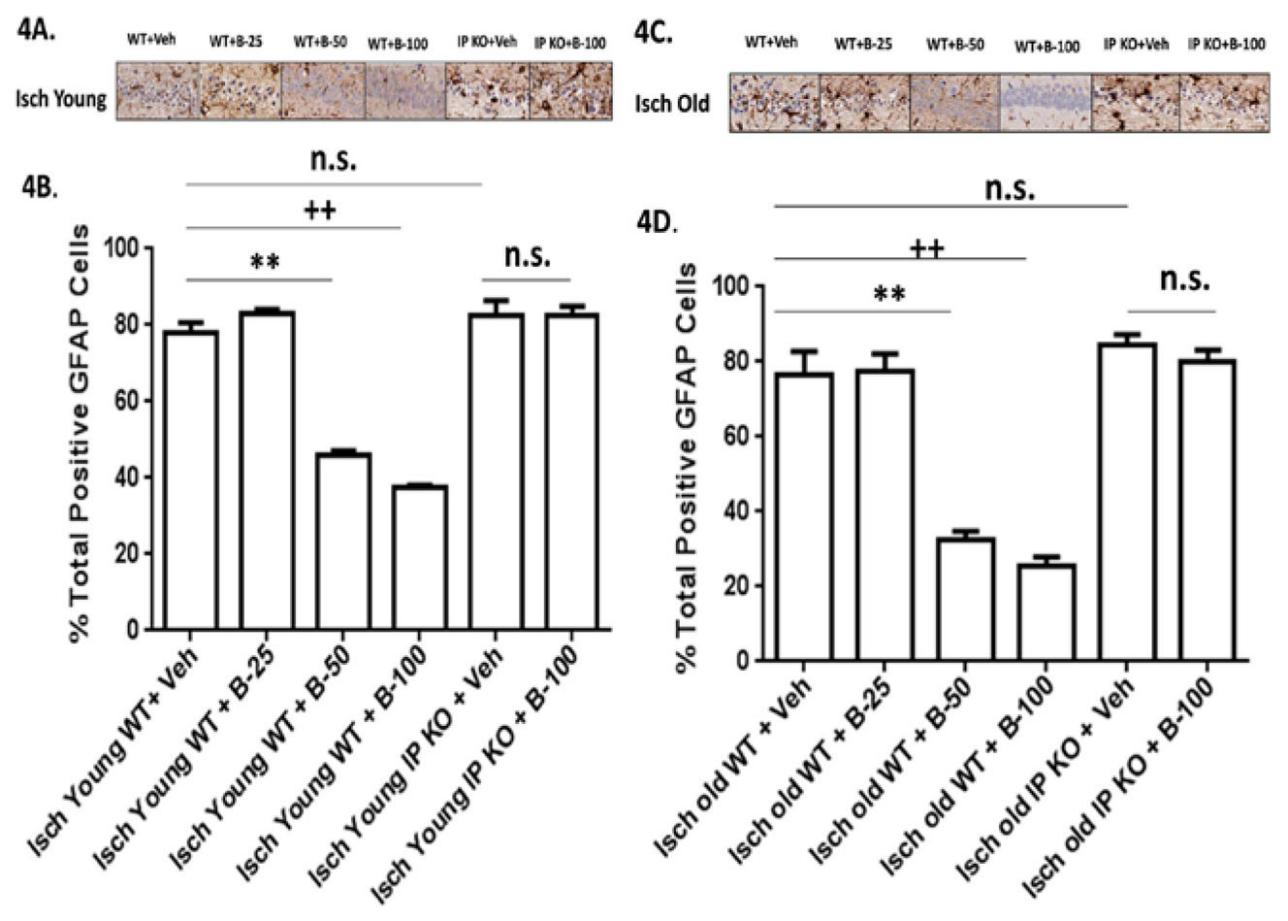
**A–D:** Post-treatment with beraprost reduces astrogliosis. (4A and 4C) Representative images of brain sections show astrocyte immunostaining in Young and Old vehicle and beraprost treated (B-25, B-50 and B-100) WT and IP KO ischemic mice. (4B and 4D). Young and Old mice were treated with beraprost (B-25, B50 and B-100) after 4 h of reperfusion and every day for 7 d following 12 min of global cerebral ischemia. Astrogliosis was assessed 7 d after ischemia and treatment with various doses of drug. n=8–10 per group. **P<0.01, ++P<0.01 vsIsch Young WT+ Veh; **P<0.01, ++ P<0.01 vsIsch Old WT+ Veh; n. s.=not significant. Data are presented as mean ± s.e.m.

**Figure 5:**
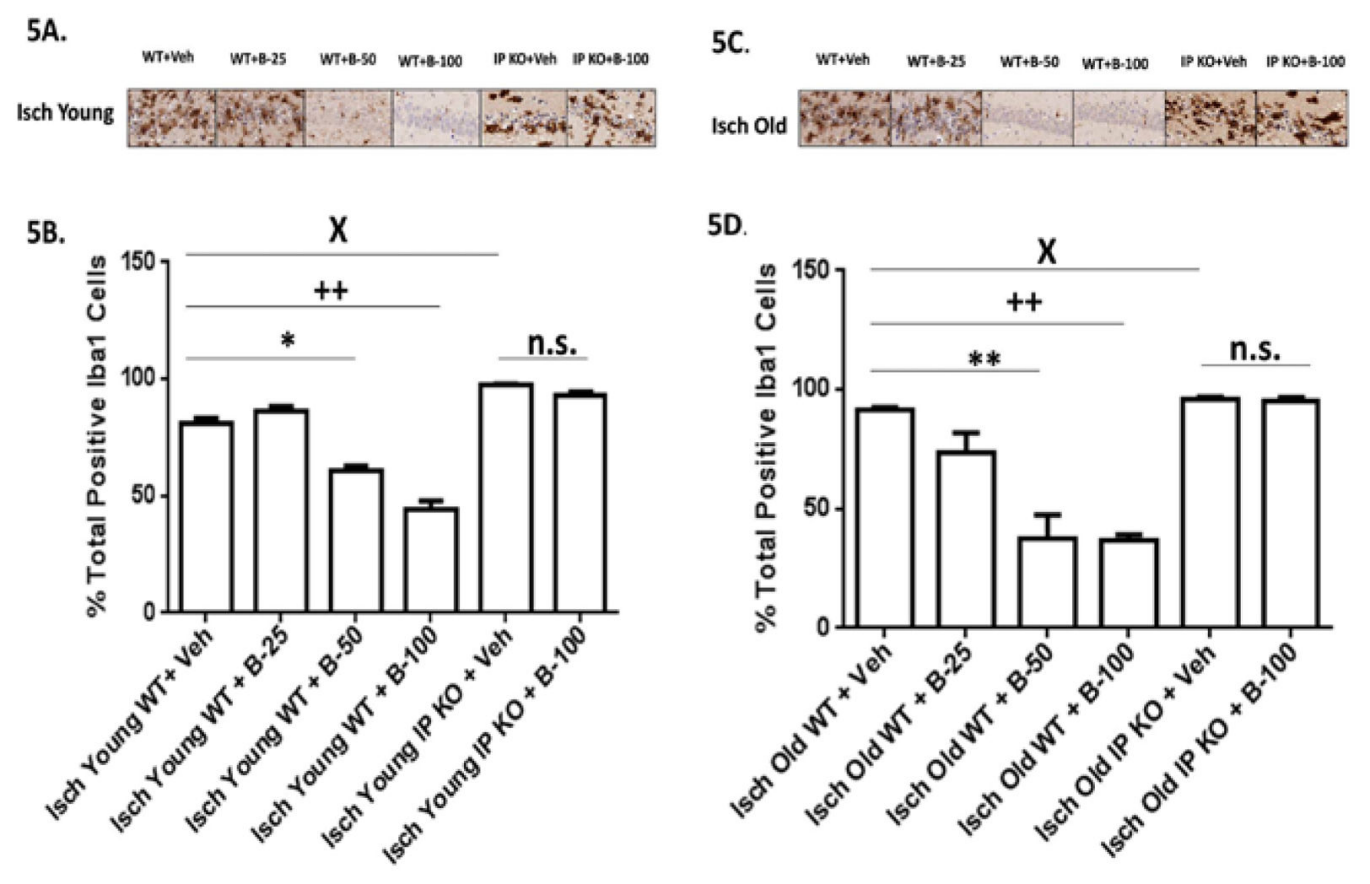
**A–D:** Microglia/macrophage invasion was reduced with beraprost treatment. (5A and 5C) Representative images of brain sections show microglia immunostaining in Young and Old vehicle and beraprost treated (B-25, B-50 and B-100) WT and IP KO ischemic mice. (5B and 5D). Active phagocytes were quantified after treatment with beraprost (B-25, B50 and B100) by counting Iba-positive cells in the hippocampal CA1 subfield on day 7 after ischemia. n=8–10per group. *P<0.05; ++P<0.01; XP<0.05 vsIsch Young WT+Veh; **P<0.01, ++P<0.01; XP<0.05 vsIsch Old WT+ Veh.; n.s.=not significant. Data are presented as mean ± s.e.m.

**Figure 6:**
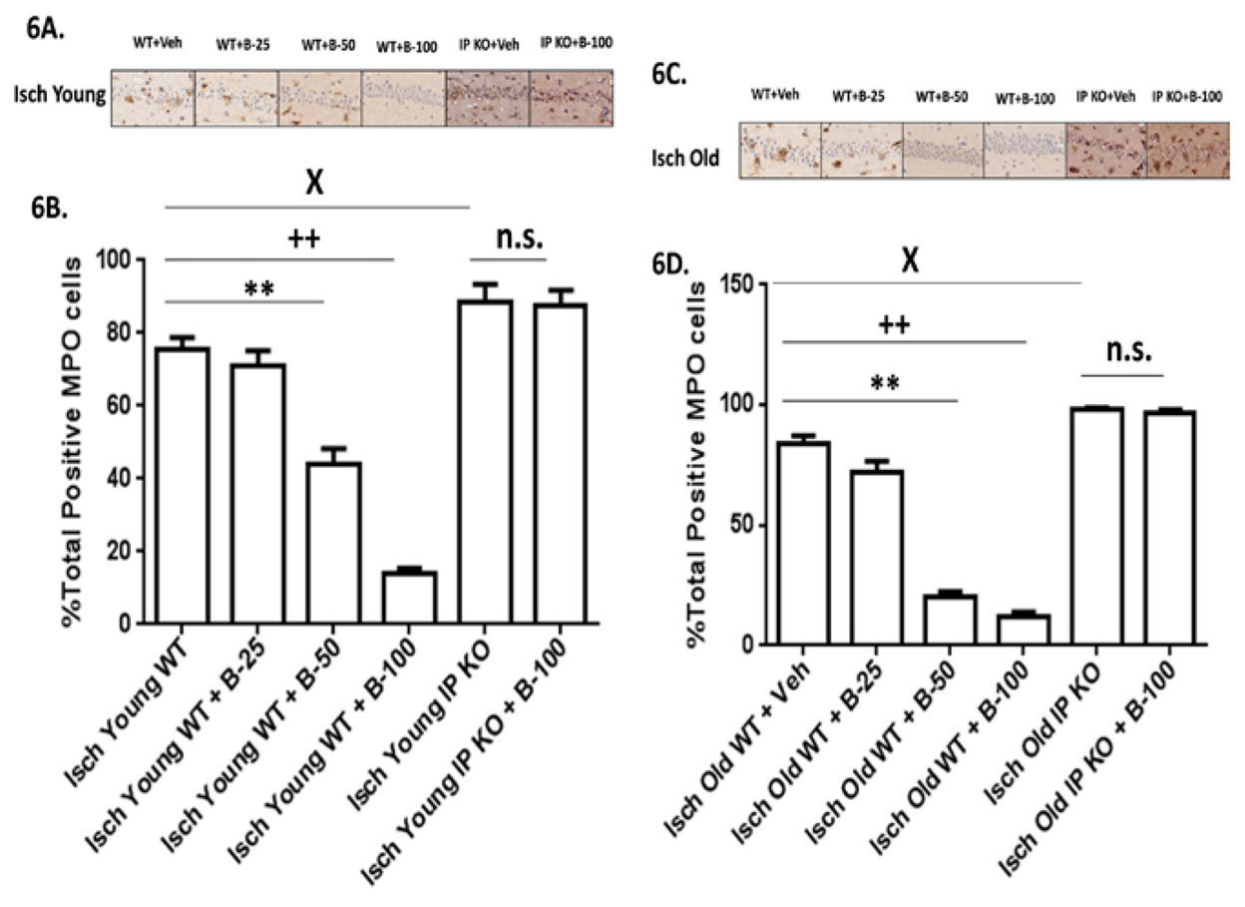
**A–D:** Neutrophil infiltration is reduced with beraprost treatment. (6A and 6C). Representative images of brain sections show MPO immunostaining in Young and Old vehicle and beraprost treated (B-25, B-50 and B-100) WT and IP KO ischemic mice. (6B and 6D). The presence of neutrophils was assessed in ischemic mice after treatment with beraprost (B-25, B50 and B-100) by quantification of MPO-positive cells in the hippocampal CA1 subfield on day 7 after ischemia. n = 8–10 per group. **P<0.01; ++P<0.01; XP<0.05 vsIsch Young WT +Veh; **P<0.01, ++P<0.01; XP<0.05 vsIsch Old WT+ Veh.; n.s.=not significant. Data are presented as mean ± s.e.m.

**Figure 7:**
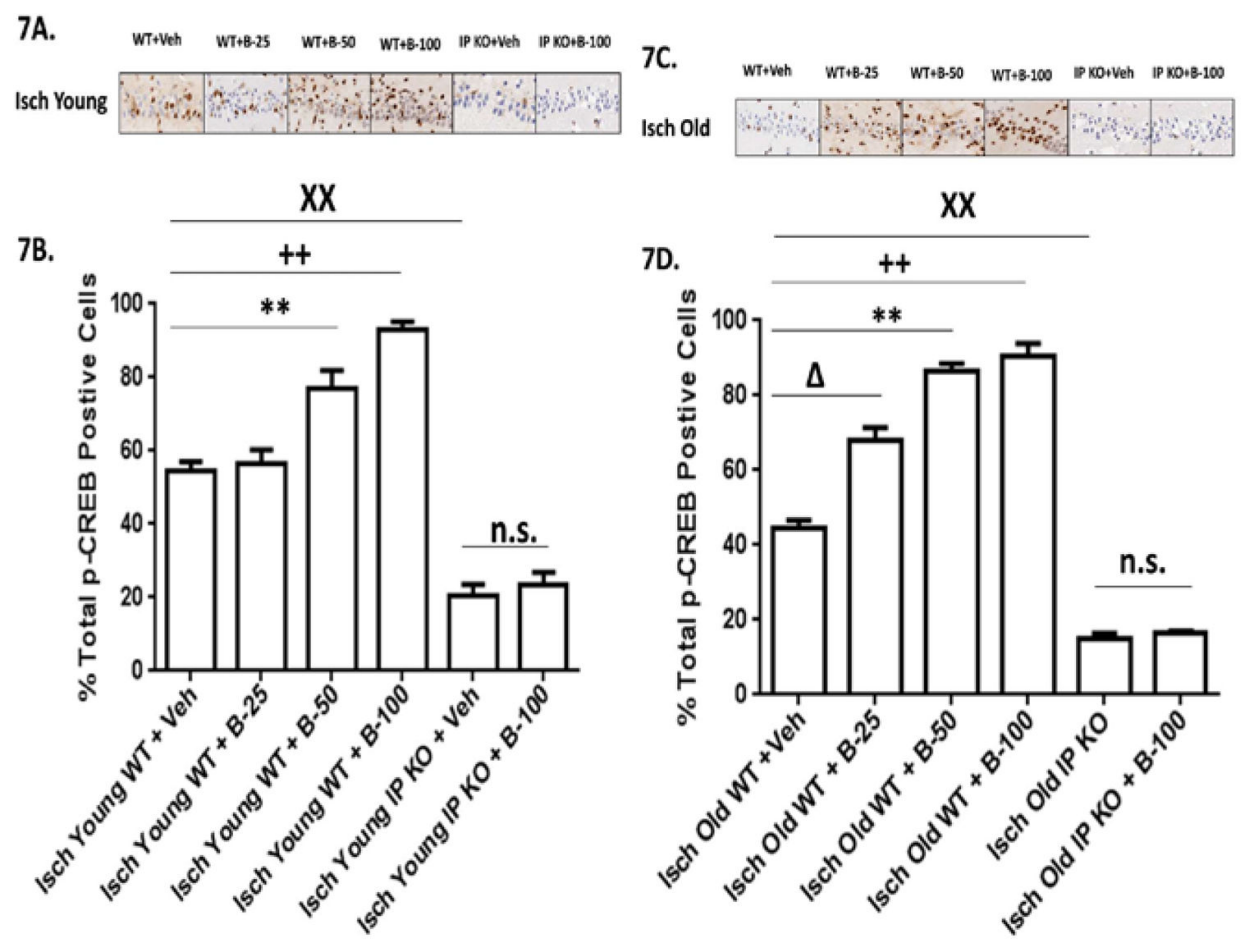
**A–D:** Phospho-CREB-positive cells are increased after beraprost treatment. (7A and 7C). Representative images of brain sections show p-CREB immunostaining in Young and Old vehicle and beraprost treated (B-25, B-50 and B-100) WT and IP KO ischemic mice. (7B,7D). Cells in the CA1 subfield of the hippocampus were stained for p-CREB after vehicle or beraprost treatment (B-25, B50 and B-100) and quantified on day 7 after ischemia. n=8–10 per group. **P<0.01; ++P<0.01, ××P<0.01 vsIsch Young WT+Veh; P<0.05; **P<0.01, ++P<0.01; XXP<0.01vs Isch Old WT+Veh; n. s.=not significant. Data are presented as mean ± s.e.m.

Moreover, the old vehicle and beraprost treated C57Bl/6 WT (B-25, B-50 and B-100) (8.9% ± 1.0%; 8.2 ± 1.1; 9.7 ± 1.0; 10.0 ± 0.5% from baseline) and vehicle IP KO and IP KO B-100 treated groups (8.1 ± 1.0; 9.0 ± 0.7% from baseline) showed drop in CBF measured by laser Doppler flowmetry during ischemia and body temperature maintained at 37.0 ± 0.5°C in all the groups. The sham groups (sham vehicle and B-100 treated young and old WT and IP KO) showed not a significant drop in CBF from baseline. Our results show a significant improvement (p<0.01) in locomotor and T-maze activity in both young (2–3 mo.) and old (12–15 mo.) ischemic WT mice as compared to analogous cohorts of IP KO mice after post treatment with beraprost but not vehicle, as shown in Figures 1A,1B,2A and 2B. At day 3, all groups showed similar trends as day 7 (data not shown). While, the sham vehicle and B-100 treated young and old WT and IP KO mice showed no significant change in loco motor activity score (5) and number of no alternations on T-maze score 3–4. Beraprost sodium (25–100 μg/kg b.w. p.o) treatment also dose dependently decreased (p<0.01) neuronal cell death, as measured by the number of cells in the hippocampal CA1 granular layer in both young and old WT mice compared to IP KO counterparts (Figure 3B and 3D). There was no significant difference in NeuN positive cells were observed in sham vehicle and B-100 treated young and old WT and IP KO mice (sham vehicle treated young WT and IP KO 88.0 ± 2.3; 90.2 ± 1.9; sham B-100 treated young WT and IP KO 91.4 ± 2.0; 84.8 ± 2.9; sham vehicle treated old WT and IP KO 88.2 ± 2.4; 84.5 ± 3.2; sham B-100 treated old WT and IP KO 89.5 ± 3.4; 82.0 ± 3.2). Astrogliosis, showed a significant decrease in WT compared to IP KO mice at the highest dose of 100 μg beraprost/kg b.w. (p<0.01). No differences were observed between young and old mice of either genotype (Figure 4B and 4D). Similarly, no astrogliosis was observed in sham vehicle and B-100 treated young and old WT and IP KO mice. Since an immune response follows upon the neuronal death caused by brain ischemia, we assessed activation of microglia and invasion of macrophages (Iba1 staining) and neutrophils, as measures of an immune response. The numbers of microglia/macrophages were reduced by beraprost treatment in a dose-dependent manner (Figure 5B and 5D). Myeloperoxidase is a marker for neutrophils. There was a significant decrease in MPO levels after post treatment with beraprost (Figure 6B and 6D). There were no microglia activation and MPO activity were observed in sham vehicle and B-100 treated young and old WT and IP KO groups. We determined levels of p-CREB since the mechanism of beraprost has been reported to involve the phosphorylation of this important transcription factor. Beraprost treatment results in the up regulated of p-CREB levels in a dose-dependent manner (p<0.01) in young and old WT mice. No significance observed in young and old IP KO vehicle or beraprost treated mice (Figure 7B and 7D). Moreover, there was no up-regulation of p-CREB levels was observed in sham vehicle and B-100 treated young and old WT and IP KO mice.

## Discussion

The present study demonstrates that post treatment of mice with beraprost following transient global cerebral ischemia induced by BCCAo markedly ameliorated hippocampal injuries as evaluated immunohistochemically and behaviorally. Moreover, the physiological and behavioral improvements were associated with a reduction of the immune response and increased CREB phosphorylation.

Several reports substantiate that transient global cerebral ischemia causes cognitive impairment [22]. The mild damage in the hippocampal CA1 region results in spatial learning deficites [23]. We likewise showed a decrease in NeuN positive cells in the CA1 region of the hippocampus in young and old WT and IP KO mice that correlates well with the impairment of behavior related to spatial preference memory, as determined by spontaneous alternations in a T-maze. Post treatment with beraprost resulted in significant improvement in cognitive function and neuronal survival in WT mice but had no effect in IP KO mice. This suggests that the PGI2 agonist beraprost, acting through the IP receptor, attenuates the cognitive functional impairments caused by BCCAo by sparing neuronal loss in the hippocampal CA1 region.

In addition, increasing evidence suggests that ischemia causes an inflammatory response characterized by astrogliosis, activation of microglia and infiltration of macrophages and neurtrophils [24,25]. Activated astrocytes, microglia/macrophages and MPO in neutrophils are the principal sources of reactive oxygen species that mediate neuronal cell damage [13,26]. Our results suggest that age is a factor in ischemic damage since older mice were more susceptible to astrogliosis, microglia/macrophage activation and MPO activity than young mice. Moreover, these increases in inflammation were dramatically reduced by beraprost treatment, suggesting that its neuro-protective activities are mediated through its anti-inflammatory properties.

COX-2 inhibitors exert harmful effects on cardiac diseases and stroke [27]. It is also reported that signaling by prostacyclin and its analogues is transduced through the IP receptor by stimulation of G protein-coupled receptors and adenylate cyclase, which leads to the enhancement of cAMP production [28]. Moreover, genetic deletion of the IP receptor reduces phosphorylation of CREB (p-CREB), which plays an important role in learning and memory [13]. Also, activation of the cAMP/PKA/CREB signaling pathway in the hippocampus plays an important role in spatial memory formation [29,30]. Our previous studies suggested that the effects of the prostanoid receptor are mediated through the cAMP/PKA pathway. Recent reports suggest that beraprost treatment prevents nephropathy and cardiac fibrosis by increasing the phosphorylation of CREB [12,31–33]. The results presented here, showing that beraprost treatment increased expression of p-CREB in CA1 hippocampal neurons after an ischemic insult in wild-type but not IP knockout mice, is consistent with this mode of action of the drug. Future work is needed to determine the complete mechanism of action of beraprost mediates through cAMP/PKA/CREB pathways after hippocampal CA1 brain injury.

## Conclusion

In summary, this is the first study suggesting that post treatment with beraprost significantly reduces cognitive dysfunction and neuronal damage caused by BCCAo. Moreover, astrogliosis, activated microglia/macrophages and MPO levels were significantly reduced and p-CREB increased in both young and old ischemic WT mice compared to ischemic IP KO mice after post treatment with beraprost. These findings show that agonists of the IP receptor can attenuate the inflammation caused by ischemia, leading to increased neuronal survival accompanied by increased expression of p-CREB, with the result that learning and memory are spared, especially in old mice.
